# From habits of attrition to modes of inclusion: enhancing the role of private practitioners in routine disease surveillance

**DOI:** 10.1186/s12913-017-2476-9

**Published:** 2017-08-25

**Authors:** Revati K. Phalkey, Carsten Butsch, Kristine Belesova, Marieke Kroll, Frauke Kraas

**Affiliations:** 1 Division of Epidemiology & Public Health, University of Nottingham, C111, Clinical Sciences Building 2, City Hospital, Hucknall Road, NG5 1PB Nottingham, Nottingham, UK; 2 Institute of Public Health, University of Heidelberg, Im Neuenheimer Feld 130.3, 69120 Heidelberg, Germany; 3 Institute of Geography, University of Cologne, Albertus-Magnus-Platz, D-50923 Cologne, Germany; 4 London School of Hygiene and Tropical Medicine (LSHTM), 15-17 Tavistock Place, WC1H 9SH, London, UK

**Keywords:** Private-practitioners participation, Disease surveillance, Barriers and facilitators

## Abstract

**Background:**

Private practitioners are the preferred first point of care in a majority of low and middle-income countries and in this position, best placed for the surveillance of diseases. However their contribution to routine surveillance data is marginal. This systematic review aims to explore evidence with regards to the role, contribution, and involvement of private practitioners in routine disease data notification. We examined the factors that determine the inclusion of, and the participation thereof of private practitioners in disease surveillance activities.

**Methods:**

Literature search was conducted using the PubMed, Web of Knowledge, WHOLIS, and WHO-IRIS databases to identify peer-reviewed and gray full-text documents in English with no limits for year of publication or study design. Forty manuscripts were reviewed.

**Results:**

The current participation of private practitioners in disease surveillance efforts is appalling. The main barriers to their participation are inadequate knowledge leading to unsatisfactory attitudes and misperceptions that influence their practices. Complicated reporting mechanisms with unclear guidelines, along with unsatisfactory attitudes on behalf of the government and surveillance program managers also contribute to the underreporting of cases. Infrastructural barriers especially the availability of computers and skilled human resources are critical to improving private sector participation in routine disease surveillance.

**Conclusion:**

The issues identified are similar to those for underreporting within the Integrated infectious Disease Surveillance and Response systems (IDSR) which collects data mainly from public healthcare facilities. We recommend that surveillance program officers should provide periodic training, supportive supervision and offer regular feedback to the practitioners from both public as well as private sectors in order to improve case notification. Governments need to take leadership and foster collaborative partnerships between the public and private sectors and most importantly exercise regulatory authority where needed.

**Electronic supplementary material:**

The online version of this article (doi:10.1186/s12913-017-2476-9) contains supplementary material, which is available to authorized users.

## Background

The 2016 outbreak of Zika virus across twenty countries in the Americas and the preceding Ebola outbreak of 2014–15 in West Africa have underlined the importance of routine disease surveillance in an increasingly interconnected world [1]. Both these epidemics also exposed the inability of the fragile public health systems within the countries to respond swiftly or to preempt the scale of the problem [2, 3].

Government spending on health is alarmingly low in a majority of low and middle-income countries, which leaves the public healthcare system chronically underfunded and impoverished [4]. Often forcing them to make compromises on the quality of care and fueling issues with patient satisfaction and acceptance [5]. Furthermore routine surge functioning over years leaves them vulnerable to breakdowns at the lowest level of imbalance. In the backdrop of these infrastructural and financial challenges within the public healthcare sector, the private sector has gained strength globally [6]. Private health service provision is significant and dominant particularly in urban areas [7, 8]. Currently, more than half of the global population lives in urban areas. This number is expected to rise to 66%^9^ as the population races to 9.7 billion by 2050 [9, 10]. We will probably witness a simultaneous and proportionate surge in both the demand and supply of private healthcare in both rural as well as urban areas globally.

Private practitioners are the preferred first point of care in emerging economies because of perceived quality, lower costs, speedy care, flexibility of payments, and accessibility [5, 11]. They already account for over 50–80% of the out and in-patient care in countries like India, China, South Africa, Brazil, and Nigeria amongst others [7, 12, 13] In their position private primary care practitioners are best placed for the early detection of outbreaks as well as routine monitoring of disease trends. However, their role in current disease control programs is largely limited to service provision and outbreak response [14]. Their contribution to health information systems is in most countries marginal and largely voluntary, leading to gross misrepresentation and underestimation of disease burdens [15, 16]. Therefore involving the private sector in routine disease surveillance is no longer a choice but a necessity.

While public-private partnerships can be cost-effective [17] and have been widely and successfully implemented in several individual disease control programs e.g. HIV/tuberculosis, polio, malaria etc. [18], their potential for routine disease surveillance remains largely untapped. The objective of this systematic literature review is to explore the experiences made across emerging economies with regard to the role, contribution, and involvement of private practitioners in disease notification. We examine the factors that determine the inclusion of and the participation thereof of private practitioners in surveillance activities.

## Methods

A systematic literature search was conducted in September and October 2015 with an additional update in February 2016 using the databases PubMed and Web of Science. Grey literature searches were conducted using WHOLIS (Library and Information Networks for Knowledge Database) WHO-IRIS (Institutional Repository for Information Sharing) and the CDC Stacks databases. The PRISMA Statement checklist for systematic reviews was referred for the review process [19].

Inclusion criteria were set at peer-reviewed, and grey full text empirical, original articles in English with no limits for year of publication or study design. The key search terms used in permutations and combinations included “*private practitioner*” (“private practice”, “private sector”, “private healthcare provider*”, “private facility*”, “non-public sector”, “non-public physician”, “for profit sector”, “for profit facility” “private physician”) and “*disease surveillance*” (“public health surveillance”, “sentinel surveillance”, “population surveillance”, “epidemiological monitoring”, “information systems”, “hospital information systems”, “health information systems”, “management information systems”, “ambulatory care information systems”, “automatic data processing”, “electronic health records”). Search algorithms always included terms related to private practice and disease surveillance (Additional file 1). Search results were merged using EndNote X7 and duplicates removed.

**Fig. 1:**
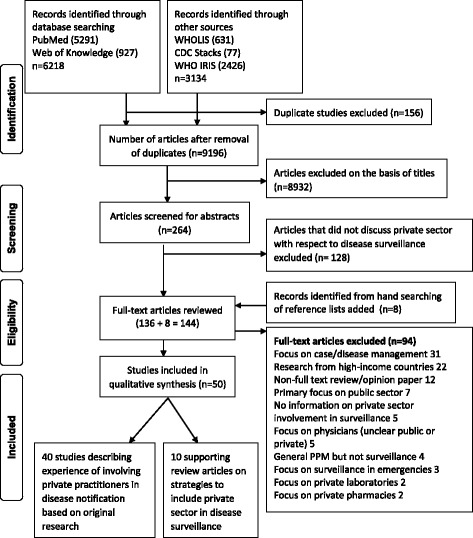
PRISMA flow diagram summarizing the literature search process

All articles (136) selected on the basis of title and abstracts were retrieved. Manual screening of reference lists identified eight further articles. A total of 144 articles were reviewed full text and data extracted by two reviewers (CB and KB) and verified independently (RP). Disagreements (13 articles) were resolved with mutual consent. Studies that explicitly investigated the role of private practitioners in routine disease surveillance were conducted in low and middle-income countries as defined by The World Bank [20] were included in the final review. The main reasons for exclusion of 94 articles (Fig. 1) included a lack of focus on routine disease surveillance, studies from high-income countries followed by non/full text opinion or review papers.

### Limitations of the review

The searches were conducted in four databases and limited to English language only which may have led to a degree of selection bias. We also used a narrow definition of private sector to include only practitioners excluding NGOs, laboratories, and pharmacies and the informal sector (unregistered or untrained) stakeholders, which limits the generalizability our findings to the private sector. We limited our search to emerging economies. Important lessons could have been drawn from experiences in the developed economies. About a third of the studies were intervention studies, which could have influenced the reporting behaviors of the practitioners. Additionally the studies were conducted in very different settings, at different scales for single and multiple diseases with both mandatory and voluntary reporting systems for different periods of observation as well as differing study designs. The heterogeneity of the studies meant that we were unable to perform in-depth analyses of the factors and could not draw generalizable inferences. For the purpose of the review we extracted data on the lessons learned and factors reported to influence reporting as identified in these studies and aim to present a summary of the facilitators and barriers to private sector involvement in routine surveillance. There were no major differences across the findings from these studies and the observations are of value in shaping the discussions and identifying specific areas of in-depth research in the future.

## Results

### Overview of the studies

Forty studies were included in the review (Table 1). In addition, we found ten review papers that provided detailed recommendations. We present them as supporting studies only (Table 2). The review summarizes the experiences drawn in 28 countries. The studies span 13 countries in Asia (Bangladesh, China, Iran, India, Indonesia, Malaysia, Myanmar, Nepal, Pakistan, Philippines, Taiwan, Thailand, and Vietnam), six in Africa (Ethiopia, Kenya, Morocco, Nigeria, South Africa, and Uganda) and nine in the South American region (Bolivia, Costa Rica, Dominican Republic, El Salvador, Guatemala, Honduras, Mexico, Nicaragua, and Peru).

**Table 1:** Overview of the studies and their main findings

No	Author, Year	Country	Scale of the study	Sample size	Response rate	Main findings and recommendations
1	Agrawal et al. 2012	Malaysia	Klang region	238 private practitioners	61%	• Implementation of an educational intervention to introduce details of pharmacovigilance into in undergraduate medical curriculum
2	Ahmadi et al. 2012	Iran	Provincial	16 disease managers for focus groups, 9 in-depth semi-structured interviews	100%	• Establishing an appropriate and simple notification process • Training human resources in disease notification • Offering incentives, privileges, and creating a positive perception of disease reporting • All solutions improve when implemented along with a proper and feasible law to determine the jurisdiction, rights, liabilities, and incentives for stakeholders
3	Ambe et al. 2005	India	City: Mumbai	All relevant providers in the RNTCP by identifying suitable roles in DOTS delivery for various providers	NA	• Coordinate involvement of private sector health care providers in an individualized manner due the heterogeneity of the sector
4	Arora et al. 2003	India	City: three areas in Delhi	200 patients for patient survey, 18 private practitioners; 101 cases for treatment outcome	Not mentioned	• Involvement of medical associations • Funding for programmes by the government • Keep it simple
5	Artawan Eka Putra et al. 2013	Indonesia	District: two districts in Bali	181 practitioners	90.5%	• Credit point system for participation • Personal contact • Continuous supervision
6	Barakat et al. 2011	Morocco	National	2007–08: 997 influenza cases and 403 severe acute respiratory illnesses; 2008–09: 1252 and 450 cases respectively	NA	• Important to include the private sector in syndromic surveillance especially when major part of care is provided by them • Even when surveillance was enhanced to include private practitioners the rate of detection remained low • Training of practitioner is necessary to improve sensitivity and specificity of diagnosis
7	Caminero & Billo 2003	South America ^a^	National	600 private practitioners	Not mentioned	• Training is the single most important factor • Work towards change of attitudes • Supervision
8	Chadha et al. 2014	India	District	8 Departments of a private medical college, 83 nursing homes, 131 peripheral health institutes; and 1766 cases	Not mentioned	• Awareness building • Government rules for case notification by private practitioners • Assistance in diagnostics and case notification, and documentation of treatment outcome
9	Chakaya et al. 2008	Kenya	City: Nairobi	46 private hospitals	57%	• Prepayment scheme as a case-holding tool
10	Chengsorn et al. 2009	Thailand	National	59 public and 26 private health care facilities and 7526 patients records.	Not mentioned	• Academic detailing’ (university-based educational outreach)
11	Chughtai et al. 2013	Pakistan	National	Number of practitioners is not mentioned	NA	• None explicit mentioned, implicitly: ensure continuous funding to support disease notification
12	Creswell et al. 2014	Pakistan	City: two cities	89 GPs and one outpatient dept. 529,447 patients	Not mentioned	• Add a new task/person or screeners in high disease burden areas
13	Daniel et al. 2013	Nigeria	State	8425 patients registered in 2011	34% in public and 1.5% private	• Provision of training and drugs for involving practitioners in a TB program (which also includes reporting activities)
14	Dowdy et al. 2013	Pakistan	City: two areas in Karachi	TB cases: 1569 (2010) pre intervention and 3140 (2011) post intervention; in the control area: 876 and 818 cases in the respective years	NA	• No recommendation on how to include private practitioners, just underlining the need to search for innovative approaches
15	Isabriye 2006	Uganda	District	109 managers, private sector providers and key informants	100%	• Ensure that all clinics and drug shops are registered and manned by qualified staff. • Identify and train nursing assistants to carry out the IDS activities (task shifting)Organize continuing professional development (CPD) courses on surveillance to improve knowledge regularly • Print and disseminate Information, Education and Communication (IEC) materials on regular basis.Regular supervision
16	John et al. 2004	India	State	NA	NA	• With participation of private practitioners district level disease surveillance system was highly successful and enabled detecting disease clustering at the start of an outbreak • Post card based disease reporting method is effective for capturing clusters of disease outbreaks • Success factors: ease of reporting, sense of contribution to the society, regular feedback through monthly disease summary bulletins
17	Khan et al. 2006	Pakistan	City: two slum areas in Lahore	5540 children 2–16 years and 5329 samples tested for microbiology	96%	• Cooperation of private practitioners is essential for complete detection of cases
18	Khan et al. 2012	Pakistan	City: two areas in Karachi	Screeners assessed 388,196 individuals at family clinics and 81,700 at Indus Hospital’s outpatient department	NA	• Engagement of intermediaries such as community members and larger hospitals as drivers of case detection • Create effective links between the public sector, private practitioners, and communities, which may include screening by community members and mass communication campaigns
19	Krishnan. 2006	India	Sub-district	146 private practitioners	72%	• Alternative healers play important role in India as private healthcare providers. • Non-involvement of the informal sector would mean large burden is missed. • They also show greater interest in working with the government, primarily because it may indirectly sanction their presence. • Involving RMPs from urban areas had more returns than from rural areas.
20	Lal 2011	India	City: 14 cities	>80,000 cases of TB	NA	• Up scaling of pp. involvement is needed; crucial: continuous mapping/registration of facilities • Continuous training with standardised material • Focus on those who expressed interest • Proactive programme officers (public health sector)
21	Lau et al. 2011	China	City: Hong Kong	247 GPs, 14 Obstetrics and Gynecology doctors and 16 Skin and Venereal Disease Specialists	27.6% for GP, 11.2% for O&B and 39.0% SVD.	• Inclusion of private practitioners in sexually transmitted disease surveillance systems can improve completeness and accuracy of reported data, which has important implications for the prevention of such diseases
22	Masjedi et al. 2007	Iran	City: Tehran	646 cases that were diagnosed as positive in the labs were followed up	NA	• Performance of the private sector should be regularly evaluated • Communications between private and public sector should be strengthened for better case notification
23	Maung et al. 2006	Myanmar	Division: Mandalay	NA	NA	• Success factors in increasing case notification through involvement of private practitioners in case notification were strong managerial support, a well-developed local medical organization, training and supervision by the public sector, and provision of free drugs and consumables
24	Naqui et al. 2012	Pakistan	City: several towns of Karachi	94 GPs from the selected towns, and 309 enrolled patients	37.50%	• Greater regulation of private practitioners to set standard guidelines • Sustained government support, and a two-way feedback mechanism from health providers necessary
25	Newell 2004	Nepal	City: Lalitpur	759 patients registered in first 24 months	67%	• Not all private practitioners need to be involved in regular surveillance. • Sentinel surveillance can work best involving larger hospitals • Provide guideline booklets
26	Palave et al. 2015	India	Sub-district: Rahata, Ahmednagar, Maharashtra	148 private practitioners	96.6% for visits/interview; 89.1% for workshop	• Strengthening of public-private partnerships through the provision of free materials, incentives, and periodic modular training in disease notification and treatment
27	Pethani et al. 201	Pakistan	City: six towns of Karachi	94 GPs, 23 Union Councils in the 6 towns. 389 patients	Not mentioned	• The use of contact screening to increase further case detection by private practitioners • Legislative approach to enforce the participation of private practitioners to participate in public-private initiatives after they have received training
28	Phalkey et al. 2015	India	City: Pune	258 private practitioners	86%	• Simplified reporting mechanisms (preferably electronic formats) • Providing clear guidelines and reporting procedures. • Organizing CMEs to strengthen practitioner knowledge and awarding CME points to those who report cases regularly are feasible solutions and should be piloted
29	Philip et al. 2015	India	District: Alappuzha, Kerala	169 private practitioners in quantitative and 34 in qualitative component	80% for quantitative; 94.4% qualitative	• Consistent motivational and attitudinal building (both private and public) to ensure compliance • Demonstrating disease notification as a mode of disease control to private practitioners • Targeting specialists in private hospitals for involvement in case notification • Behavioural changes such as timely dissemination of policy changes, and soft skills training, and improvement of interpersonal skills • Involvement of a liaison officer dedicated to public-private coordination
30	Portero et al. 2003	Philippines	National	1355 private practitioners	57.9%	• Awareness building among private practitioners (responsibility) • Establish a network with well-trained practitioners • Establish clear treatment and referral structures (also from private to public sector in the case of TB)
31	Quy et al. 2003	Vietnam	City: 22 districts of Ho Chi Minh City	30 practitioners	96.6%	• Involvement of private practitioners through training and distribution of referral forms • Introduction of financial incentives for private practitioners • Supervision of private practitioners
32	Rangan et al. 2003	India	City: Mumbai	NA	NA	Improvement of the quality of care, e.g., through training in patient - health care provider interaction
33	Sarkar et al. 2012	India	Sub-district: Alipurduar, Jalpaiguri, West Bengal	6191 cases of malaria; 336 cases of severe malaria	NA	• Further research to identify the reasons for under reporting (burden of paper work, unfamiliarity with notifiable diseases, etc.) • An annual review of case records at facilities to identify unreported deaths and enhance completeness of reporting
34	Shinde et al. 2012	India	City: seven health posts of municipal ward, Mumbai	104 private medical practitioners (PMP)	Not mentioned	• Greater emphasis by public health agencies on legal and public health basis for reporting conditions • Training private practitioners to report the presumptive as well as confirmed cases of diseases under surveillance • Use of appropriate software for paperless communication in case reporting • Encourage the use of standard the prescribed formats for reporting by private practitioners • Provision of private practitioners with periodic telephonic communication and alert messages regarding notification
35	Singh et al. 2015a	South Africa	National	NA	NA	• Considerable education and relationship building exercises necessary • Stakeholder consultation essential for common understanding and shared vision • Large hospitals more compliant than independent practitioners • Despite legislation reporting is poor • Absence of electronic data biggest challenge • Peer networking e.g. Senior Oncologist to champion the cause of case reporting •
36	Srivastava et al. 2011	India	District: Gwalior	200 allopathic private practitioners	Not mentioned	• Regular upgrade in knowledge • Provision of additional benefits to the private practitioners to increase the rates of notification
37	Tan et al. 2009	Taiwan	National	15 of 26 counties/cities selected, 1093 private practitioners	87.4%	• Modify doctor’s attitude to disease reporting • Developing a convenient and widely-accepted reporting system (phone reporting where possible) • Establishing reward/penalty system essential in improving reporting compliance in private doctors.
38	Yeole et al. 2015	India	City: Pimpri Chinchwad Municipal Corporation(PCMC) area, Pune	831 for the quantitative, 24 for qualitative	64% for quantitative and 100% qualitative	• Provision of training for private practitioners • Targeted media communication campaigns • Establish alternative mechanisms for notification (to facilitate notification), e.g., internet and mobile telephones, to save the time spent on notification
39	Yimer et al. 2012	Ethiopia	Region: Amhara	112 private practitioners	77%	• Regular training • Feedback and mutual information between private sector and referral institutions in the public sector
40	Zafar Ullah et al. 2012	Bangladesh	City: four areas in Dhakacity; later scaled up to twomajor cities	97 PMPs in 2004, 703 at the end of 2009	100%	• Provision of training • Provision of tools and protocols • Mutual trust

^a^Mexico, El Salvador, Honduras, Guatemala, Nicaragua, Peru, Dominican Republic, Costa Rica, Bolivia

**Table 2:** Overview of the supporting studies

No	Author, Year	Country	Main findings and recommendations
1	Arora and Gupta, 2002	India	• Formats for record keeping at a private health facility should be simple and concise • Laboratories should be identified within or in the vicinity of private health facilities. • Expertise enhanced via appropriate training programs • Multiple awareness-campaigns are necessary • State must provide incessant administrative and a financial support to both public and private sectors
2	Chitkara et al. 2013	India	• Limited awareness in private sector with regards to reporting • Interaction with professional bodies • Stronger collaboration between governments and professional bodies and the private sector • Sensitizing private sector through professional body meetings • Dissemination of information through professional body publications • Online reporting platform • Integrated voice recording and SMS reporting • Inculcate confidence
3	Dewan et al. 2006	India	• Private sector involvement in surveillance is feasible and cost-effective • Professional societies such as Indian Medical Association are essential partners in bringing together public and private sectors • Advocacy, training, supervision are necessary to maintain interest of the private sector
4	Kirsch & Harvey, 1994	Global	• Private practitioners fail to report cases because of ignorance on reporting requirements and procedures • Patient confidentiality • Perceptions that reporting is time consuming, motivation and excessive workloads • Reasons are similar to public sector • Underfunding, under staffing and lack of supervision main determinants for under reporting • Remove obstacles to reporting • Only relevant data to be collected, checklist rather than forms • Clear contact person • Incentives in form of recognition from professional bodies, free laboratory reagents, journal subscriptions etc.
5	Lei et al. 2015	Global	• Multiple collaborative mechanisms promote case detection, confirmation and reporting • Incentives e.g. free tests and drugs are useful approach to improve private sector participation • Regulations should be enforced punishment for non reporting also adopted • Compulsory to improve the knowledge, consciousness and behavior of the practitioners • Training courses should enforce an exam that needs to be passed • Better governance from the program managers and the government • Lack of communication and mistrust reduces mutual understanding between the public and private sectors
6	Revankar, 2004	India	• Simplify guidelines • Provide technical assistance to the practitioners • Provide financial assistance for capacity building within the private sector • Establishing partnerships is difficult sustaining them even bigger challenge • Heavy inputs from the governments necessary • Motivation and interests of the private practitioners difficult to monitor and sustain
7	Nagaraja et al. 2014	Global	• Raise awareness amongst private practitioners regarding surveillance • Regular media campaigns and advocacy to sustain interest • Strong regulatory and punitive action for non reporting • Including private sector without legal back up is difficult • Governments should consider providing infrastructural support such as handheld devices • Use friendly and simple notification as per practitioner preference • Varied reporting formats to be accepted simple web based application, SMA, toll free number or paper based reporting as per practitioner preference
8	Uplekar et al. 2001	Global	• Sensitization of the private sector essential • Training is essential to improve case detection, confirmation and notification • Signing of Memorandum of Understanding (MOU) and letter of agreements • Concerted efforts sustained overtime necessary
9	Uplekar, 2003	Global	• Improved role for the government in providing information, regulation and financing of trainings for private sector • Revamping UG and PG medical curricula to enhance record keeping practices • Teaching hospitals as essential links between public and private sectors • Telephone line and onsite visits for trouble shooting • Bilateral visits to understand the work of the other (public and private sectors) • Mutual respect, working through consensus and inclusion of private sector in policy making can improve compliance • Problems identified ✓ Public sector: lack of will to take on private sector, preoccupied with several programs, believe eventually patients will come to them, little common ground for collaboration with heterogeneous, unregulated private sector, in the absence of regulation view them as unmanageable ✓ Private sector: absence of information, do not agree with national guidelines as they ate not a part of making them, critical of distrust shown towards them, reluctant to loose their patients
10	WHO, 2015	Global	• NGOs and private labs are useful intermediary institutions • Public and private partnerships can be win-win partnerships for all stakeholder when implemented well • Include private sector on discussion boards of national committees • Monthly face to face meetings build trust • Mandatory regulations to ensure compliance • Professional bodies and role models can be used to improve practitioner reporting

All the studies except one [21] were single country studies conducted between 1996 and 2015 and a majority of them were from India (15, 37.5%) [14, 22–37]. The scale of the studies varied from small city areas to the national levels, but about half (19, 47.5%) of them were conducted at an individual city level [11, 16, 17, 22, 23, 28, 31, 33, 37–46].

Fifteen (37.5%) studies were mixed methods surveys. Eleven (27.5%) were intervention studies targeted at improving private practitioners participation in disease control programs [11, 17, 21, 22, 28, 29, 40, 41, 47–49]. Nine studies (22.5%) used secondary record review to evaluate disease notification, and four (10%) of them were Knowledge Attitude and Practice (KAP) surveys. The average private practitioner response rate (in 20 studies) was 78% and ranged from 1.5% in Nigeria [50] to 100% in Iran [51], Bangladesh [46], and Uganda [52]. Three studies from India [29, 30, 37] reported a higher response rate to interviews compared to requests of record reviews.

All except one study [34] referred to communicable diseases (STIs, malaria, typhoid, influenza and five studies [26, 33, 51–53] with multiple diseases) and a majority (28, 70%) were related to tuberculosis. Eighteen studies (45%) refereed to laboratory confirmed case detection and ten (25%) referred to both suspected as well as laboratory confirmed cases. Only one addressed syndromic surveillance for influenza [54]. Eight studies (20%) [15, 16, 27, 29–31, 52, 54] investigated private practitioners reporting behaviors towards voluntary case reporting. Thirteen (32.5%) [17, 24, 28, 34, 37, 38, 40, 41, 44, 50, 53, 55, 56] investigated systems that expected mandatory case reporting from the private sector.

Fifteen studies (15, 37.5%) [16, 22, 25, 27, 28, 34, 38, 40, 43, 45, 46, 48, 55, 57, 58] stated that involving private sector reduced diagnostic delays and improved case detection (7 to 50%). Even when only a fraction of private practitioners became active, the case detection rose significantly [22]. Involving the private practitioners in surveillance activities also helped identify an emerging disease (leptospirosis) in India [26], recognize patterns in health seeking behaviors in China [42], Nigeria [50], and Morocco [54] and detect comorbidities in Kenya [38].

### Barriers to notification

Eight (20%) [15, 16, 30, 33, 37, 49, 51, 53] studies identified knowledge of the practitioner as the most important determinant of case detection, confirmation and notification (Table 3A). Knowledge about disease control programs and their diagnostic requirements was higher amongst public as compared to private practitioners [36]. Although the knowledge regarding disease detection was lower amongst alternate medicine practitioners as compared to allopath practitioners in India, the surveillance practices did not differ significantly [14]. General practitioners were more likely to be aware about the importance of notification than specialists in India [29, 30] and Malaysia [15]. The duration (>5 years) of practice (OR 11.4, 95% CI 1.99, 65.58, *p* = 0.001) was significantly associated with practitioner reporting in Uganda [52]. In Malaysia, practitioners were willing to report cases only when they were confident of their diagnosis [15].

**Table 3:** Barriers to case reporting at the practitioner and government/public sector end as identified by the studies

A	Barriers to reporting: practitioner end	n %
1	Knowledge	
	Lack of information what, how, where to report /unfamiliarity on reporting process/system [15, 16, 30, 33, 37, 49, 51, 53]	8 (20%)
2	Attitudes	
	Motivation [11, 14, 51, 59] and lethargy [15]	5(12.5%)
	Should be financially reimbursed [15, 27]	2 (5%)
	Disease reporting not considered a priority [51]	1 (2.5%)
3	Perceptions	
	Patient confidentiality [14, 15, 30, 34, 37, 53, 56]	8 (20%)
	Legal issues [14, 15, 33, 51]	4 (10%)
	Complicated reporting systems [37, 51, 53]	3 (7.5%)
	Fear of losing patients [16, 28]	2 (5%)
	Beyond scope of clinicians responsibilities /No obvious benefit [11, 51]	2 (5%)
	Misconception about reporting procedures [30]	1 (2.5%)
	Appear foolish if misdiagnosed [15]	1 (2.5%)
	Fear notification may trigger further investigations [15]	1 (2.5%)
4	Practice	
	Infrastructure issues such as human (adequate and skilled, staff turnover) resources and equipment resources [14, 17, 27, 31, 32, 48, 50, 51, 58]	9 (22.5%)
	Lack of time/additional burden [14–17, 33, 34, 53]	7 (17.5%)
	Lack of reporting forms/registers [15, 37] impractical design [15]	3 (7.5%)
	No lab or technician [11, 26, 48]	3 (7.5%)
	Cost of reporting [59]	1 (2.5%)
B	Barriers to reporting: government and public sector end	n %
	Lack of clear instructions/inadequate dissemination of guidelines/no assistance with reporting procedures, supervision or feedback etc. [14, 22, 24, 33, 34, 43, 46, 48, 51]	9 (22.5%)
	Lack of cooperation/coordination/collaborative environment/positive dialogue (Govt. and private sectors) [14, 22, 30, 37, 46, 48, 51]	7 (17.5%)
	Lack of leadership/strong and proactive administration [28, 31, 34, 48, 55, 59]	6 (15%)
	No punitive action or regulation [11, 22, 30, 53] (separate regulatory function from service provision [27])	5 (12.5%)
	Non-involvement of range of private healthcare providers [11, 22, 27]	3 (7.5%)
	Other (Red tapism [33], lack of simplified system [37]	2 (5%)

Apart from disease knowledge a clear understanding of the notification procedures is critical to reporting. Lack of clear instructions, inadequate dissemination of guidelines and no assistance with reporting procedures, supervision or feedback were identified as the most important reasons for under reporting in nine studies (22.5%) [14, 22, 24, 33, 34, 43, 46, 48, 51] (Table 3B). Over 50% of the 238 practitioners in the study from Malaysia did not know whom to report to and did not have reporting forms [15]. In Uganda about half (49%) of the 109 practitioners knew where to send the report but only 21% (*n* = 23) knew which form to use [52]. Practitioners who considered the system inconvenient or were unfamiliar with the reporting procedures were less likely to report cases in Taiwan [53].

Simplicity of reporting procedures and the mode of reporting offered (telephone, email, paper etc.) were important determinants in India [33] and Taiwan [53]. Findings from Bangladesh [46] and India [37] indicate that reporting compliance increases over time as the familiarity to the system grows and therefore sustained efforts are necessary. The rated public health importance of the disease (e.g. cholera > enteric fever) or sense of emergency (e.g. Zika) also affects disease notification from the private practitioners [26].

Appreciating the importance of surveillance or adequate knowledge of the disease and its reporting procedures does not ensure case notification. Two studies in India suggested that only about half of the practitioners who understood the importance of notification agreed to report/participate in surveillance activities [30, 33]. Yeole et al. (2015) support these findings [37] where although 64% of 831 practitioners agreed to participate in a surveillance system, only 16% (*n* = 87) actually notified data. Although about half of the participants (104, 46%) in a study in Mumbai said that there were no barriers to reporting, this was not reflected in their reporting practices [33].

The attitude of the practitioners was reported as significant determinant of case notification in studies from Malaysia [15], India [36], and Uganda [52].

Of the 238 practitioners in a study in Malaysia, 73.1% exhibited unsatisfactory “attitude” towards disease notification, 81.9% showed complacency, 66.9% ignorance and 23.5% indifference respectively [15]. Ullah et al. (2012) report it was the negative attitudes of the practitioners towards the government officials rather than the notification process itself that affected reporting in Bangladesh [46]. Ahmadi et al. (2013) note that it was the negative attitude of the data collectors towards data compilation and towards the practitioners, which discouraged practitioners from notification in Iran [51].

Lal et al. (2011) and Ambe et al. (2005) conclude that there is mutual distrust and prejudices and suggest that trust building is necessary at both the public and private practitioners end alike. Seven (17.5%) studies [14, 22, 30, 37, 46, 48, 51] and six (15%) studies [28, 31, 34, 48, 55, 59] identified the lack of coordination/collaboration between the government and the private sector as the main barrier for case reporting respectively (Table 3B). There is a need for managing perceptional conflicts at both ends [30]. While the government sector should attract and sustain private practitioner attention, the private practitioners should exhibit their responsibility towards disease notification [22, 28]. Five (12.5) studies [11, 14, 15, 51, 59] suggest that the motivation of the practitioners also played an important role in disease notification.

While eight studies (20%) [14, 15, 30, 34, 37, 53, 56] and four (10%) studies [14, 15, 33, 51] identified breach of patient confidentiality and legal issues as reasons for not reporting respectively, Philip et al. (2015) suggest this is a perception only as legal frameworks in most countries allow case notification to governments [30].

Nine (22.5%) studies [14, 17, 27, 31, 32, 48, 50, 51, 58] reported the lack of adequate and skilled staff and equipment (e.g. computers) as the main barriers to case reporting. Interestingly only seven (17.5%) studies [14–17, 33, 34, 53] identified lack of time as the main barrier. While the maintenance of records within individual facility was significant determinant of case notification in Ethiopia [60], the availability of information materials, registers and reporting formats affected case reporting in Uganda [52]. Access to a laboratory (OR 3.79, 95% CI 0.99, 14.55, *p* = 0.05) played an important role in the willingness of private practitioners to report cases in India [17].

### Recommendations to improve reporting

Obtaining an overview of the private sector by identifying the different actors and clearly stating their roles and responsibilities was recommended as the first step towards government regulation of the private sector (Table 4) with regard to disease surveillance [11, 22, 27, 28, 31, 45, 52]. Krishnan et al. (2006) suggest a separation of the governments’ regulatory function from public service provision to ensure strict action against reporting defaulters [27]. Eight studies each recommended standardization of unified reporting procedures [11, 22, 31, 33, 46, 48, 51, 57] and earmarked public financial resources for capacity building within the private sector for disease surveillance [14, 17, 21, 23, 31, 40, 52, 55].

**Table 4:** Recommendations to improve private practitioner participation in disease surveillance

	National government level	No of Studies (%)
1	Registration and regulation of the private sector [11, 22, 28, 31, 45, 52] Involvement of wide range of healthcare providers [11, 22, 27]	9 (22.5%)
2	Standardized reporting procedures with roles and responsibilities clearly stated [11, 22, 31, 33, 46, 51, 57] Unified recording and reporting system [48]	8 (20%)
3	Financial (earmarked funds for private sector) and human resource assistance from public sector [17, 31, 52, 55] and funds for training [14, 21, 23, 40]	8 (20%)
4	Establish surveillance legislation/legal frameworks [17, 33, 34] Stakeholder consultation in policy making [34, 42, 46]	6 (15%)
5	Credit point system for participation [33, 36, 49] Comparable measurable performance indicators and audits [30, 32, 51]	6 (15%)
6	UG and PG medical curricula [15, 28, 29, 51, 57]	5 (12.5%)
7	Mandatory notification [14, 26, 30]	3 (7.5%)
	District and local administrative level	
1	Staff and practitioner training [14, 15, 21, 25, 28–31, 33, 34, 36, 46, 48–52, 55, 57, 60] CME [14, 29, 30, 36, 37]	25 (62.5%)
2	Dissemination of information, IEC materials, mass communication campaigns [16, 29, 31, 34, 36, 37, 40, 50, 52, 57, 60]	11 (27.5%)
3	Establish and strengthen formal collaborations [27, 45] Communication with practitioners [30, 32, 33, 43] Leadership [59] Strong and proactive administration [28, 48]	9 (22.5%)
4	Simplified reporting procedure [14, 32, 33, 37, 51, 53, 55]	7 (17.5%)
5	IMA (interface organizations assistance) [25, 29, 31, 48, 55]	5 (12.5%)
6	Provide reporting forms/appropriate softwares [33, 45, 49] [26]	4 (10%)
	Surveillance program level	
1	Feedback (summary bulletins, review meetings etc.) [11, 16, 17, 26, 27, 31, 45, 46, 52] Acknowledgement of efforts [14]	10 (25%)
2	Supportive supervision and visits onsite [25, 27, 33, 45, 46, 48, 49, 51, 52]	9 (22.5%)
3	Financial incentives [16, 17, 27, 29, 40, 51] Reward and penalty system [53] Non cash incentives [14]	8 (20%)
4	Allow diverse reporting mechanisms to overcome perceived barriers [11, 30, 33, 37, 53, 58]	6 (15%)
5	Technical assistance [31, 45] Laboratory services [24, 45, 49]	5 (12.5%)
6	Personal contact/ relationship and trust building [27, 33, 34, 37, 46]	5 (12.5%)

At the district administration level, the main recommendation was to provide surveillance training (regular [14, 15, 21, 25, 28–31, 33, 34, 36, 46, 48–52, 55, 57, 60] and continuing medical education [14, 29, 30, 36, 37]). Five (12.5%) studies recommended revision of regular undergraduate and postgraduate medical curricula to incorporate a stronger focus on surveillance activities [15, 28, 29, 51, 57]. Eleven (27.5%) studies [16, 29, 31, 34, 36, 37, 40, 50, 52, 57, 60] recommended that IEC materials and guidelines should be widely disseminated. Eleven (27.5%) studies [27, 30, 32–34, 37, 43, 45, 46] recommended improving the communication between the government, public and private sectors (Table 4).

At the program level, feedback (10, 25% studies) [11, 14, 16, 17, 26, 27, 31, 45, 46, 52] and supportive supervision (9, 22.5% studies) [25, 27, 33, 45, 46, 48, 49, 51, 52] were the main recommendations. Eight studies (20%) [14, 16, 17, 27, 29, 40, 51, 53] recommended that in the absence of regulation there may be the need to provide an incentive/reward (e.g. CME credits) for reporting. Only one study suggested that the incentives could be non-financial (e.g. technical assistance, supervision, free diagnostics etc.) [14, 18].

## Discussion

Findings of the review suggest that the knowledge, attitudes, perceptions, and practices of the government, the public sector practitioners (who implement surveillance programs), and the private sector practitioners affect case notification. Interventions targeting all three groups are therefore mandated. Training, timely feedback, and regular supportive supervision from the program managers can improve case notification. Simple standardized reporting procedures with clear guidelines and effective communication between those providing and receiving the data are key to effective private practitioner participation in disease surveillance. More importantly regular publication of disease data from both public and private sectors can encourage reporting as it serves as feedback to those reporting. Improved representativeness serves as evidence base and aids the use of data for decision making at the national levels.

The factors affecting case reporting from the private practitioners as identified in this review are similar to those reported for under reporting from the public sector within the Integrated Disease Surveillance and Response systems (IDSR) which collect routine data mainly from the public sector [61, 62]. Therefore an important first step would be to make the distinction between the roles of the policy makers, healthcare providers and people (individual practitioners)! There is the urgent need to separate the words public sector and government [27]. While public sector facilities and practitioners funded through general taxes are responsible for implementing disease control programs in most LMICs, the role of the government is wider. Governments bear the responsibility to regulate and develop both the public as well as the private sectors equally [5]. All data collection should be substantiated with follow up action in order to encourage reporting compliance in both the public and private sectors. Furthermore involvement of the end users (patients and communities), non-government and civil advocacy organizations should be considered to create enabling environments for disease data reporting.

Heterogeneity of private sector [22, 45] and the varying size, arrangements and functionality of the informal sectors [40] are probably the main reasons why it is a resource and logistical challenge to regulate the private sector in most LMICs [31]. Often records of *all* healthcare providers are not available even with the government which is a challenge for assessing reporting consistency and disease burdens [14]. Governments should exercise authority and use registration as a tool to regulate the formal private sector facilities as a start. Although troublesome to implement, a “single registration platform” for all providers should be advocated to record the legal status, size, and nature of services provided [63]. Stronger legislative and regulatory frameworks are necessary to harmonize the diverse set of stakeholders [6, 17].

The next step would be to simplify and standardize the reporting procedures, provide clear guidelines and ensuring that the information reaches every intended private practitioner [18, 64]. Where possible, data should be obtained in a format (email, toll free number, text-messages, paper forms etc.) preferable to the practitioner and without incurring extra work or interrupting workflows [33]. Private sector should be involved in decision-making processes with regards to surveillance and invited to become members of national policy implementation boards [13, 65].

Human resource development in the private sector should be seen as important as in the public sector. Governments should invest in the training of private practitioners (CME, workshops etc.) and accessory staff in surveillance procedures. Medical curricula should be revisited to emphasize the importance of reporting [13]. A substantial “knowledge-application/practice gap” exists and even amongst practitioners who know and understand the importance of reporting only about a half will notify [17]. Therefore intermittent sensitization campaigns highlighting the importance and processes of surveillance are useful [66]. Small individual clinics constitute a major proportion of the private sector and the public sector should have sufficient capacity to train and supervise the large (and growing) numbers [59].

In addition, supportive supervision through onsite visits by program officers facilitate trust building and should be implemented [67]. While this is resource intensive, extensive initial input followed by regular follow up pays off in the long run [37, 46]. “Without feedback, practitioners soon realize that it makes no difference whether they report. Information is of value only when it is used for decision-making” [68]. Regular feedback (telephone, newsletter, review meetings etc.) is an essential determinant of provider motivation and should be mandatorily provided [69–71].

Four [35, 72–74] studies suggested that reporting should be mandatory and a penalty be imposed for non-reporting. However voluntary systems are more accurate and although legal obligation evokes reporting, reporting is more complete when the practitioner understands the importance of sending reports [75, 76]. Six studies [16, 17, 27, 29, 40, 51] from India, Iran and Pakistan suggested that in the absence of regulatory frameworks financial incentives may become necessary for consistency in voluntary reporting. While there are fundamental differences in the values of individuals in the not for profit public and the only for profit private sectors that are difficult to navigate, this approach is not sustainable [77]. Awarding non-financial incentives such as free training, credit points towards Continuing Medical Education (CME) for consistent reporting, free drugs, laboratory tests, and access to scientific journals or books etc. may be offered. Infrastructure assistance such as software loaded basic handheld devices with a direct reporting tools could be an option for the future [5, 66]. Alternately commissioning surveillance from large private hospitals with closely monitored contracts and performance-based incentives may prove beneficial [6]. Once routine data reporting improves, efforts to investigate the quality of the data received from private sector and comparative assessments between public and private providers could be useful.

The links between the governments, public, and private sectors need to be identified and strengthened. Intermediary bodies such as Non-Governmental Organizations [25, 28, 31], professional associations [25, 64], private labs [35, 78], private medical colleges [13], and pharmacies [73, 74] are crucial connectors and can facilitate communication between the three groups [35, 72–74]. Practitioners in LMICs often work in both the public and private sectors. Those with dual roles could be roped in to initiate a dialogue with the private sector and also promote peer reporting from defaulting practitioners [65]. Practitioners’ ranking based on consistency in routine disease data reporting has been attempted within the Integrated infectious Disease Surveillance and Response system (IDSR) in India and could be tested at scale in other settings. Most importantly all actors need to acknowledge the overarching role of the government to regulate and support them at the same time. Sharing of knowledge and best practices alongside skills transfer should be encouraged across regional and national governments in order to avoid delays in implementing changes already tried and successful in better involvement of the private sector in routine disease surveillance.

## Conclusion

The current private practitioner participation in disease surveillance efforts is appalling. The main barriers to their participation are inadequate knowledge leading to unsatisfactory attitudes and misperceptions that influence their practices. Complicated reporting mechanisms with unclear guidelines along with unsatisfactory attitudes on behalf of the government also contribute to the under reporting. Infrastructural barriers such as availability of computers and human resources need rectification. Governments need to take leadership and foster collaborative partnerships between public and private sectors for routine disease surveillance and exercise authority when needed. Surveillance program officers need to provide periodic training, offer supportive supervision and regular feedback to practitioners from both public as well as private sectors.

## Additional file

Additional file 1: Provides an overview of the search strategy and search terms by databases used for the review. (DOCX 140 kb)

## Abbreviations

CDC: Centers for Disease Control
CME: Continuing Medical Education
DFG: Deutsches Forschungsgemeinschaft (German Research Foundation)
LMICs: Low and Middle Income Countries
NGOs: Non Government Organizations
OR: Odds Ratio
WHO IRIN: WHO Global Digital library
WHOLIS: WHO Library database
