# Supplementary material for: From habits of attrition to modes of inclusion: enhancing the role of private practitioners in routine disease surveillance
Source: BMC Health Serv Res. 2017 Aug 25;17:599. doi: 10.1186/s12913-017-2476-9 (PMC5574140; doi:10.1186/s12913-017-2476-9)
Supplement: Additional file 1: — Provides an overview of the search strategy and search terms by databases used for the review. (DOCX 140 kb) [file 12913_2017_2476_MOESM1_ESM.docx]

| **PUBMED** | |
| --- | --- |
| #1 | Disease surveillance[All Fields] AND "private"[All Fields] |
| #2 | surveillance and "private practitioners" |
| #3 | ("epidemiology" [Subheading] OR "Public Health Surveillance"[Mesh] OR "Sentinel Surveillance"[Mesh] OR "Population Surveillance"[Mesh] OR "Epidemiological Monitoring"[Mesh] OR "Epidemiology"[Mesh]) AND ("Private Sector"[Mesh] OR "Hospitals, Private"[Mesh] OR "Private Practice"[Mesh] OR "Public-Private Sector Partnerships"[Mesh] ) |
| #4 | ((private practitioner[All Fields] OR private practitioners[All Fields]) OR (private practice[All Fields] OR private practiceinternational[All Fields] OR private practices[All Fields]) OR (private sector[All Fields] OR private sector's[All Fields] OR private sectors[All Fields]) OR private sector providers[All Fields] OR (private provider[All Fields] OR private providers[All Fields]) OR private health providers[All Fields] OR (("patients' rooms"[MeSH Terms] OR ("patients'"[All Fields] AND "rooms"[All Fields]) OR "patients' rooms"[All Fields] OR "private"[All Fields]) AND ("delivery of health care"[MeSH Terms] OR ("delivery"[All Fields] AND "health"[All Fields] AND "care"[All Fields]) OR "delivery of health care"[All Fields] OR ("health"[All Fields] AND "care"[All Fields]) OR "health care"[All Fields])) OR (private doctor[All Fields] OR private doctors[All Fields]) OR (private physician[All Fields] OR private physicians[All Fields]) OR (private facilities[All Fields] OR private facility[All Fields]) OR (private clinic[All Fields] OR private clinical[All Fields] OR private clinician[All Fields] OR private clinicians[All Fields] OR private clinics[All Fields]) OR (private hospital[All Fields] OR private hospitalists[All Fields] OR private hospitalization[All Fields] OR private hospitalizations[All Fields] OR private hospitals[All Fields])) AND "disease Surveillance"[All Fields] |
| #5 | ("private practitioner"[All Fields] OR "private practice"[All Fields] OR "private sector"[All Fields] OR "private provider"[All Fields] OR (("patients' rooms"[MeSH Terms] OR ("patients'"[All Fields] AND "rooms"[All Fields]) OR "patients' rooms"[All Fields] OR "private"[All Fields]) AND ("health"[MeSH Terms] OR "health"[All Fields]) AND ("Provider"[Journal] OR "provider"[All Fields] OR "IHS Prim Care Provid"[Journal] OR "provider"[All Fields])) OR "private health care"[All Fields] OR "private doctor*"[All Fields] OR "private physician"[All Fields] OR "private facility"[All Fields] OR "private clinic"[All Fields] OR "private hospital"[All Fields] OR (non-public[All Fields] AND ("Practitioner"[Journal] OR "practitioner"[All Fields] OR "JK Pract"[Journal] OR "practitioner"[All Fields])) OR (non-public[All Fields] AND ("Practice (Birm)"[Journal] OR "practice"[All Fields])) OR "non-public sector"[All Fields] OR (non-public[All Fields] AND ("Provider"[Journal] OR "provider"[All Fields] OR "IHS Prim Care Provid"[Journal] OR "provider"[All Fields])) OR (non-public[All Fields] AND ("health"[MeSH Terms] OR "health"[All Fields]) AND ("Provider"[Journal] OR "provider"[All Fields] OR "IHS Prim Care Provid"[Journal] OR "provider"[All Fields])) OR "non-public health care"[All Fields] OR (non-public[All Fields] AND ("physicians"[MeSH Terms] OR "physicians"[All Fields] OR "doctor"[All Fields])) OR (non-public[All Fields] AND ("physicians"[MeSH Terms] OR "physicians"[All Fields] OR "physician"[All Fields])) OR (non-public[All Fields] AND facility[All Fields]) OR (non-public[All Fields] AND ("ambulatory care facilities"[MeSH Terms] OR ("ambulatory"[All Fields] AND "care"[All Fields] AND "facilities"[All Fields]) OR "ambulatory care facilities"[All Fields] OR "clinic"[All Fields])) OR (non-public[All Fields] AND ("hospitals"[MeSH Terms] OR "hospitals"[All Fields] OR "hospital"[All Fields]))) AND ("epidemiology"[Subheading] OR "Public Health Surveillance"[Mesh] OR "Sentinel Surveillance"[Mesh] OR "Population Surveillance"[Mesh] OR "Epidemiological Monitoring"[Mesh] OR "Epidemiology"[Mesh]) |
| #6 | surveillance system AND "private" |
| #7 | ("private") AND "case detection" |
| #8 | ("private") AND "case reporting" |
| #9 | ("private") AND "disease data reporting" |

| **Web of Knowledge** | |
| --- | --- |
| #1 | "private sector" or "private practitioner" AND "disease surveillance system*" |
| #2 | ("epidemiology" [Subheading] OR "Public Health Surveillance"[Mesh] OR "Sentinel Surveillance"[Mesh] OR "Population Surveillance"[Mesh] OR "Epidemiological Monitoring"[Mesh] OR "Epidemiology"[Mesh]) AND ("Private Sector"[Mesh] OR "Hospitals, Private"[Mesh] OR "Private Practice"[Mesh] OR "Public-Private Sector Partnerships"[Mesh] ) |
| #3 | TOPIC: ("Public Health Surveillance" OR "Population Surveillance") AND TOPIC: ("private sector" or "private practitioner*" or "public-private partnership*") |
| #4 | "disease Surveillance") AND TOPIC: ("private") |
| #5 | TOPIC: ("case reporting") AND TOPIC: ("private") |
| #6 | **TOPIC:** ("case notification") *AND* **TOPIC:** ("private") |
| #7 | **TOPIC:** ("case detection") *AND* **TOPIC:** ("private") |
| #8 | **TOPIC:** ("surveillance system*") *AND* **TOPIC:** ("private") |

| **CDC Stacks** | |
| --- | --- |
| #1 | "Private Practitioners" And "Surveillance" |
| #2 | " All Fields Contains ""Case Reporting"" AND All Fields Contains "Private" " |
| #3 | " All Fields Contains ""Disease Surveillance"" AND All Fields Contains "Private |
| #4 | " All Fields Contains ""Data Reporting"" AND All Fields Contains ""Private"" |
| #5 | " All Fields Contains ""Surveillance"" AND All Fields Contains ""Private"" " |
| #6 | All Fields Contains ""Disease Surveillance"" AND All Fields Contains ""Private Sector"" |
| #7 | "Disease Data Reporting" AND All Fields Contains "Private" " |
| #8 | All Fields Contains ""Private Sector"" AND All Fields Contains ""Surveillance System"" " |

| **WHOLIS** | |
| --- | --- |
| #1 | words or phrase "surveillance" AND words or phrase "private" |
| #2 | words or phrase ""disease surveillance"" AND words or phrase "private" |
| #3 | words or phrase ""reporting"" AND words or phrase "private" |
| #4 | words or phrase "report" AND words or phrase "private" |

| **WHO IRIN** | |
| --- | --- |
| #1 | private and "surveillance" |
| #2 | private and “disease reporting” |
| #3 | “private practitioner” and surveillance |
| #4 | “PPM” and surveillance |
